# A case report on therapeutic anticoagulation in the management of a COVID-19 patient on antiplatelets post-CABG — how much is enough?

**DOI:** 10.1186/s42077-023-00333-7

**Published:** 2023-05-30

**Authors:** Geetanjali Tolia Chilkoti, Kushal Thakkar, Pallav Bhandari, Medha Mohta, Deepti Agarwal

**Affiliations:** Department of Anesthesiology and Critical Care, University College of Medical Sciences and Guru Teg Bahadur Hospital, Shahdara, Delhi, 95 India

**Keywords:** Coronavirus disease, Antiplatelets, Anticoagulants

## Abstract

**Background:**

Coronavirus disease (COVID-19) predisposes patients to both arterial and venous thrombosis due to excessive inflammation, platelet activation, endothelial dysfunction, and stasis. Therefore, therapeutic anticoagulant therapy has been an integral part for management in patients with moderate to severe COVID-19 disease. We shall here discuss the concerns of starting anticoagulants in patients with COVID-19 disease who are already on antiplatelets, an unexplored area.

**Case presentation:**

We herein report a case of 61-year-old patient with hypertension and diabetes mellitus type 2 with COVID-19 disease. Patient also had coronary artery disease and underwent CABG 4 years ago and was on aspirin 75-mg HS, clopidogrel 75-mg OD, and tab atorvastatin 40-mg HS. Patient was hemodynamically stable and was maintaining a saturation of 66% on room air and a saturation of 96% on 0_2_ by high FiO_2_ partial rebreathing face mask. On auscultation, crepitations were present in bilateral lower lung fields. The patient was admitted under moderate category of COVID-19 SARS in the intensive care unit (ICU). Despite of standard care and treatment, in next 2 days, the oxygenation deteriorated Pa02/Fi02 < 200, and intermittent noninvasive ventilation had to be started, and patient’s clinical condition fell into the severe disease category, wherein there was a need to start IV methylprednisolone and to start the therapeutic dose of anticoagulant, i.e., enoxaparin. The D-dimer was greater than 1000 ng/mL therapeutic dose of enoxaparin, i.e., 2 U/kg in two divided doses was started. Patient’s condition improved. In the present case, we fraught with the increased risk of bleeding or mortality subsequent to increasing the dose of anticoagulant as the patient was already on antiplatelets.

**Conclusions:**

We emphasize that one must evaluate the risk and benefit of bleeding vs improved oxygenation prior to considering the therapeutic dose of LMW heparin in patients who are already on antiplatelets. A track on d-dimer and fibrinogen levels for dose titration may be useful step in this set of patients. We reiterate upon the formulation of dogmatic guidelines in this context is warranted.

## Background

Coronavirus disease (COVID-19) predisposes patients to both arterial and venous thrombosis due to excessive inflammation, platelet activation, endothelial dysfunction, and stasis. Therefore, therapeutic anticoagulant therapy has been an integral part for management in patients with moderate to severe COVID-19 disease. We shall here discuss the concerns of starting anticoagulants in patients with COVID-19 disease who are already on antiplatelets, an unexplored area.

### Case presentation

A 61-year-old male patient, weighing 94 kg (BMI 34), presented in the first week of May 2021 with chief complaints of fever, shortness of breath, and dry cough for the past 1 week, and RT-PCR was tested positive. Patient is known case of hypertension for the past 26 years and newly diagnosed case of diabetes mellitus and was on tab enalapril 2.5-mg BD and tab metformin 1-gm OD. Patient also had coronary artery disease and underwent CABG 4 years ago and was on aspirin 75-mg HS, clopidogrel 75-mg OD, and tab atorvastatin 40-mg HS. On presentation in hospital, patient had a pulse rate of 101/min, BP of 150/79 mm Hg, and respiratory rate of 38 breaths/min. The patient was maintaining a saturation of 66% on room air and a saturation of 96% on non-rebreathing face mask (NRBM) on 15 L/m of flow. On auscultation, crepitations were present in bilateral lower lung fields. No abnormal heart sounds were audible on auscultation. Laboratory investigations were normal except for high WBC count. A 12-lead ECG revealed left axis deviation, and chest X-ray revealed bilateral lower zone infiltrates. The patient was admitted under moderate category of COVID-19-SARS in the intensive care unit (ICU) and managed as per the clinical guidance by ICMR (AIIMS/ICMR-COVID-19 protocol n.d.)). Inj. meropenem, inj. vancomycin, ivermectin, doxycycline, hydroxychloroquine, enoxaparin, dexamethasone, pantoprazole, vitamin C, multivitamins, and self-proning were started. He remained hemodynamically stable and maintained oxygenation (maintained P/F ratio > 200 on NRBM with 15 L/m flow) for the next 2 days. On day 3 of ICU, his oxygenation deteriorated suddenly (*SpO2* < 80 mmHg on NRBM and Pa02/Fi02 < 200) and hypotension (*BP* < 90 mmHg despite hydration). Chest X-ray was repeated, and pneumothorax was ruled out, and a clinical diagnosis of pulmonary embolism was suspected. Intermittent noninvasive ventilation was started, and patient’s clinical condition fell into the severe disease category, wherein IV methylprednisolone, a 1 mg/kg in two divided doses and therapeutic dose of anticoagulant, i.e., enoxaparin, i.e., 2 U/kg in two divided doses, was started. The D-dimer at this time point was greater than 1000 ng/mL, and lower limb venography was suggestive of deep venous thrombosis in deep peroneal and superficial veins. The facility for computed tomography-pulmonary angiography was not available; however, a clinical diagnosis of pulmonary embolism was made. Patient’s condition improved in next few days.

Coronavirus disease 2019 (COVID-19) is caused by the severe acute respiratory syndrome coronavirus 2 (SARS-CoV-2) which may predispose patients to both arterial and venous thrombosis due to excessive inflammation, platelet activation, endothelial dysfunction, and stasis. Therefore, prophylactic dose of LMW heparin is an integral part in all COVID-19 patients requiring hospital admission; for patients with a contraindication to pharmacologic prophylaxis, mechanical prophylaxis should be used (Thachil et al. 2020, Bikdeli et al. 2020). As far as its therapeutic use is concerned, an observational study showed that therapeutic anticoagulation was associated with decreased mortality in patients with COVID-19 who required mechanical ventilation, but not in all hospitalized patients with COVID-19 (Paranjpe et al. 2020). Patients requiring mechanical ventilation who were treated with therapeutic anticoagulation had an inhospital mortality of 29.1% compared to 62.7% in patients who did not receive anticoagulation. Longer duration of anticoagulation was associated with a reduced risk of mortality (Paranjpe et al. 2020).

However, in the present case, we encountered a patient who was already on antiplatelets following CABG which raised the associated concern of increasing the dose of anticoagulants following the fall of patient to the severe disease category. We fraught with the increased risk of bleeding or mortality subsequent to increasing the dose of anticoagulant as the patient was already on antiplatelets.

We used d-dimer levels as the guide for anticoagulation in this case. The elevation of D-dimer in severe cases reflects pulmonary thrombosis. Moreover, it has been well documented that abnormal D-dimer is helpful in indicating deep venous thrombosis in cardiovascular diseases (Gao et al. 2020) Sivaloganathan et al. concluded that patients taking either of the antithrombotic therapy, i.e., anticoagulant or antiplatelet agents at the time of infection with COVID-19, do not have a significantly different mortality risk to those patients not taking these drugs. The observations were not statistically significant due to the limited sample size. However, they did not consider the patients who were on both antiplatelets and anticoagulants like our patient (Sivaloganathan et al. 2020).

## Conclusions

The risk and benefit of bleeding vs improved oxygenation must be evaluated prior to considering the therapeutic dose of LMW heparin in patients who are already on antiplatelets by simultaneously keeping a track on d-dimer and fibrinogen levels in patients with severe COVID-19 disease.

## Data Availability

Not applicable.

## Abbreviations

COVID: Coronavirus disease 2019
SARS-CoV-2: Severe acute respiratory syndrome coronavirus 2
ICU: Intensive care unit
